# Amalgams for the Teeth

**Published:** 1875-04

**Authors:** E. A. Bogue


### SELECTED ARTICLES.

### ARTICLE V.

### *Amalgams for the Teeth.*

BY E. A. BOGUE, M. D.

The physical properties of amalgams are as yet but little understood; and we are glad to know that experiments upon them are now being made, both here and in England, which we trust, will result in valuable additions to our knowledge.

Meanwhile, for information on this subject, we are obliged to rely mainly upon Watts' Chemical Dictionary; and for information concerning amalgams for dental use, we are largely indebted to Messrs. Tomes and Fletcher, of England, and Cutler and Beers, of America.

The alloys for dental amalgam are various. Though mostly compounded of silver and tin, yet gold, platinum, palladium, cadmium, antimony, and copper have been sparingly used.

Some of these metals combine with mercury easily; others with difficulty.

Among the former are silver, gold, tin and cadmium. Among the latter, copper, palladium, and platinum.

It may be well to observe, in passing, that copper combines with mercury by being precipitated upon it; palladium readily, even violently assimilates with it, when in form of a precipitate, though very slowly in any other form; and platinum enters into combination as platinum sponge, or in some alloys, though Dr. Cutler (*Can. Jour. Den. Sc.*, Sept. 1871, p. 324) is not aware that this metal can amalgamate with mercury.

As to the character of these amalgams:

The copper amalgam, which is quite difficult to prepare, is dark in color, and its oxide is poisonous. Though extensively used in Germany, it is fortunately seldom or never employed in this country.

The palladium amalgam is quite as black as the copper, and cannot be used except in the form of a precipitate; but it is not liable to more than superficial oxidation, and that entirely innoxious.

While most metals combined with mercury are supposed to constitute merely mechanical admixtures or solutions, palladium forms a true chemical union attended by the evolution of heat.

This amalgam is very highly esteemed in certain quarters in England; but its costliness, blackness, and brittleness, however, prevent its being generally adopted.

The amalgams which have been most in favor in this country have been, until within a few years past, composed principally of silver and tin.

Among these, however, are some that exhibit traces of other metals; such as Townsend's and Lawrence's, and perhaps a few others, which contain the copper that enters into the composition of the coin silver they use, and Holmes' which contain a small proportion of gold that is added to diminish the shrinkage.

Recently, through the researches of Messrs. Fletcher and Tomes, a new amalgam alloy has been introduced, which is composed of gold, platinum, silver and tin. The office of the silver is to harden; of the gold, to lessen contraction and oxidation; of the platinum, to hasten the setting and preserve the color. All these are essential points, and more essential in England, perhaps than elsewhere, because an amalgam filling there, as a rule, receives its entire finish at the time of its insertion.

This filling hardens more rapidily than any other which has been in common use; and its contraction is less than that of any other *compound* filling whose contractions have been accurately measured.

As to the shrinkage: It has been thought by some that the action of certain amalgams in the tooth, drawing together and rising toward the centre, was indicative of expansion. But it may be accounted for by the tendency of some metals to assume the form of a spheroid; and the amalgams which harden most slowly are especially inclined to this shape.

Besides, the experiments which have been made upon amalgams by the specific gravity test, have exhibited marked shrinkage.

According to a paper read by Mr. Tomes before the Odontological Society of Great Britain, March 4th, 1872, the variation is from .037 of a unit in the case of palladium to .38 of a unit in the case of tin and silver used in equal parts; the shrinkage amounting to ten times more in the latter case than in the former.

The shrinkage of copper also is very little—scarcely greater than that of palladium.

It has been claimed, however, that the specific gravity test is not sufficiently accurate to measure the shrinkage of these substances; but a mechanical apparatus, constructed for the purpose of testing the very minute expansion and contraction of bodies, furnishes substantially the same results.

We wish now to speak of the practical use of amalgams. It is the general practice to combine the alloy with an excess of mercury; afterwards squeezing out the surplus mercury with the fingers or a pair of pliers.

As it is impossible to get rid of the mercury by this operation, since about twice the necessary quantity remains, leaving the amalgam hard and unworkable, the only proper course is to use the exact proportion necessary to the combination. Should a surplus of mercury at any time be found on the surface of an amalgam filling when the packing is finished, it can be tolerably well absorbed by slices of crystal gold, cut thin with a razor and laid upon the dry surface of the filling, until they are white with the mercury—when they are removed

Dr. Cutler, in his experiments, took twenty-four grains of amalgam, whose composition is not given. and having mixed, pressed, washed, and weighed in the usual way, found that thirteen grains of mercury had been retained. Further on, in the same article, he states that twenty-four grains of coin fillings took up thirty-two grains of mercury·

A red heat for twenty minutes drove off nearly all the mercury, but did not soften the lump, which remained hard and firm, though somewhat brittle. In these cases the proportions are not correct.

Now, if chemically pure silver and tin be combined in atomic proportions, silver 108, tin 118 (*vide* Fletcher on Amalgams—*Brit. Jour.*, 1872, p. 89,) twenty-four grains of the clean filings mixed with seven grains of mercury "will result in a powder, adhesive under pressure which will not dissolve in alcohol, and therefore needs no washing, and which will weld up as solid as a coin."

"This is a true amalgam, containing no free mercury; in fact, there is great difficulty in separating a trace of mercury below a red heat."

But, of course, it is impossible to use a powder in the majority of cases.

But there is a filling which it is practicable to use in almost all circumstances, viz: the ordinary silver and tin amalgam mentioned above, with the addition of ten per cent. of fine gold and sufficient platinum to insure rapid setting.

If to twelve grains of alloy four or five grains of mercury be added, and the resulting compound be carefully packed, without washing, into the cavity, little by little, with small points, warmed, if necessary, and finished up by repeated burnishing, the result will be a more perfect filling than can be procured by ordinary means, and that, too, with a compound containing little or no free mercury.

From what has been said, it will be seen that the term amalgam has been applied indiscriminately to almost all sorts of compounds; more than two hundred of which have been tested in order to ascertain their physical properties and adaptability to dental purposes.

And it is desirable, in the circumstances, that much more should be known in regard to these compounds, before they be generally adopted or rejected by the profession.—*Dental Miscellany.*

